# Differences in Peak Oxygen Uptake in Bicycle Exercise Test Caused by Body Positions: A Meta-Analysis

**DOI:** 10.3389/fcvm.2021.734687

**Published:** 2021-10-11

**Authors:** Xiaohua Wan, Chang Liu, Thomas P. Olson, Xiankun Chen, Weihui Lu, Wei Jiang

**Affiliations:** ^1^Department of Cardiology, Guangdong Provincial Hospital of Chinese Medicine, Guangzhou, China; ^2^The Second Affiliated Hospital of Guangzhou University of Chinese Medicine, Guangzhou, China; ^3^Division of Cardiovascular Diseases, Department of Internal Medicine, Mayo Clinic and Foundation, Rochester, MN, United States; ^4^Healthy Systems and Policy, Department of Global Public Health, Karolinska Institutet, Stockholm, Sweden; ^5^Key Unit of Methodology in Clinical Research, Guangdong Provincial Hospital of Chinese Medicine, Guangzhou, China; ^6^Guangdong Provincial Key Laboratory of Chinese Medicine for Prevention and Treatment of Refractory Chronic Diseases, Guangzhou, China

**Keywords:** cardiopulmonary exercise test, peak oxygen uptake, bicycle, posture difference, meta-analysis

## Abstract

**Background:** As demand for cardiopulmonary exercise test using a supine position has increased, so have the testing options. However, it remains uncertain whether the existing evaluation criteria for the upright position are suitable for the supine position. The purpose of this meta-analysis is to compare the differences in peak oxygen uptake (VO_2peak_) between upright and supine lower extremity bicycle exercise.

**Methods:** We searched PubMed, Web Of Science and Embase from inception to March 27, 2021. Self-control studies comparing VO_2peak_ between upright and supine were included. The quality of the included studies was assessed using a checklist adapted from published papers in this field. The effect of posture on VO_2peak_ was pooled using random/fixed effects model.

**Results:** This meta-analysis included 32 self-control studies, involving 546 participants (63% were male). 21 studies included only healthy people, 9 studies included patients with cardiopulmonary disease, and 2 studies included both the healthy and cardiopulmonary patients. In terms of study quality, most of the studies (*n* = 21, 66%) describe the exercise protocol, and we judged theVO_2peak_ to be valid in 26 (81%) studies. Meta-analysis showed that the upright VO_2peak_ exceeded the supine VO_2peak_ [relative VO_2peak_: mean difference (MD) 2.63 ml/kg/min, 95% confidence interval (CI) 1.66-3.59, *I*^2^ = 56%, *p* < 0.05; absolute VO_2peak_: MD 0.18 L/min, 95% CI 0.10-0.26, *I*^2^ = 63%, *p* < 0.05). Moreover, subgroup analysis showed there was more pooled difference in healthy people (4.04 ml/kg/min or 0.22 L/min) than in cardiopulmonary patients (1.03 ml/kg/min or 0.12 L/min).

**Conclusion:** VO_2peak_ in the upright position is higher than that in supine position. However, whether this difference has clinical significance needs further verification.

**Systematic Review Registration:** identifier, CRD42021233468.

## Introduction

The cardiopulmonary exercise test (CPET) is a non-invasive and safe method for comprehensive evaluation of cardiopulmonary function during exercise. It has been used in a variety of settings including differential diagnosis, surgical risk assessment, and prognosis evaluation. For example, patients with heart failure and chronic obstructive pulmonary disease with low peak oxygen uptake (VO_2peak_) have low survival rates (1, 2).

The two most common modes of CPET are upright bicycle and treadmill, followed by supine bicycle. Because of less arm and torso movement during supine cycling compared to upright cycle or treadmill, there can be less artifact in collected metrics and greater ease in obtaining clear and stable cardiac imaging and circulatory measurements when patients are supine. Therefore, supine CPET combined with cardiac imaging is the most comprehensive and sensitive means to evaluate the state of cardiac chambers, cardiac hemodynamics, and valve function during exercise (3, 4). Due to this advantage, clinical demand for supine CPET has been on the rise. However, researchers have hypothesized that the two positions' CPET results may be different. This is because the change from the upright position to supine position will affect the venous return, cardiac output (CO), the lung ventilation/perfusion matching (V/Q), and skeletal muscle blood flow and perfusion (5–8).

Studies have compared the cardiovascular response between upright and supine cycle exercise tests, but the results have been inconsistent. For example, Kramer's study of 14 men with heart failure showed that the VO_2peak_ in the upright position exceeded that in the supine (9). Conversely, Bonzheim's study found that VO_2peak_ in the supine was slightly higher than that in upright in patients with coronary artery disease (10). Therefore, the objective of this study was to compare the VO_2peak_ attained from upright and supine lower extremity bicycle exercise.

## Methods

This systematic review was conducted and reported in accordance with the Preferred Reporting Items for Systematic Reviews and Meta-Analyses statement (Supplementary Materials S1: PRISMA 2020 Checklist) and the Cochrane Handbook for Interventional Reviews and registered in PROSPERO (CRD42021233468).

### Data Sources and Search Strategy

We searched PubMed, Web Of Science and Embase on March 27, 2021 using relevant keywords and a Boolean search string (see Supplementary Materials S2 for the detailed search strategy): (((((“Exercise test”)) OR (“exercise test” OR “cardiopulmonary exercise test” OR “cycle exercise” OR “ergometer” OR “CPET” OR “CPX”)) AND (“position” OR “posture” OR “supine” OR “recumbent” OR “recline” OR “lean” OR “tilt” OR “clinostatism” OR “decubitus” OR “lie”)) AND (“erect” OR “upright” OR “orthostatic” OR “sit”)) AND (“VO_2_” OR “oxygen uptake”). The search string consisted of MeSH and general search terms. Searches were restricted to English.

### Inclusion and Exclusion Criteria

We included self-control trials, and selection criteria conformed to the PICOS approach, as described hereinafter.

#### Populations

There were no restrictions regarding subjects, except for persons with disabilities.

#### Intervention

Subjects completed an incremental maximum exercise test using a leg bicycle ergometer in a supine position. However, we excluded any studies if other interventions had been applied, such as the use of drugs that may affect hemodynamics, or lower limb negative pressure.

#### Comparators

The same subjects completed the exercise using a leg bicycle ergometer in an upright position with the same exercise protocol.

#### Outcomes

The outcome measure was absolute and/or relative VO_2peak_.

### Study Selection

After eliminating duplicate articles, XW and CL screened the titles and abstracts. Studies that did not mention VO_2peak_ or a synonym in the study title and/or abstracts, but were likely to have included them as a secondary measure, were also included. In the second step, XW and CL read the full texts of articles considered relevant based on title and abstract. There was full agreement on the inclusion of the full-text articles.

### Assessing Methodological Quality

XW and CL assessed the quality of the included studies with a modified version of the Downs and Black checklist (see Supplementary Materials S3) (11). This checklist has been employed in several reviews in the field of sports science, which also uses cross-sectional studies for data retrieval. In our modified version, we considered four domains, including seven items, to evaluate the included studies' quality: (1) are the interventions of interest clarified? (2) are the test positions clarified? (3) is the time period the participants have between tests similar? (4) was the test order randomized? (5) how was VO_2peak_ defined? (6) was the VO_2peak_ test valid? and (7) are the characteristics of the participants included in the study clarified? All items were rated as “Yes,” “No,” or “Not sure.”

### Data Extraction

We extracted data on VO_2peak_ in the respective positions and the characteristics of the participants (number of participants, sex, age, body mass, types of disease) as well as the starting workload, rotation rate, duration and workload increase of the increments used during the test protocols.

### Statistical Analysis

We performed statistical analysis with RevMan version 5.3. The data are segmented or combined based on the angle between the upper body and the horizontal plane, or whether there is cardiopulmonary disease, and the calculation process is based on the formula in Cochrane 7.7. Continuous variables are expressed by means and standard deviation. We assessed heterogeneity across included studies using a Cochran chi-square test (with 0.1 as the cutoff for statistical significance) and an *I*^2^ statistic test. We employed a random effects model where there was evidence of statistical heterogeneity (*I*^2^ statistic > 50%). Otherwise, we used a fixed effects model. If there was heterogeneity, we first analyzed the source of heterogeneity, and then used subgroup analysis or other methods to deal with it. After excluding obvious clinical heterogeneity, we conducted the meta-analysis with a random response model. The meta-analysis test level was *p* = 0.05.

## Results

### Search Results

**Figure 1:**
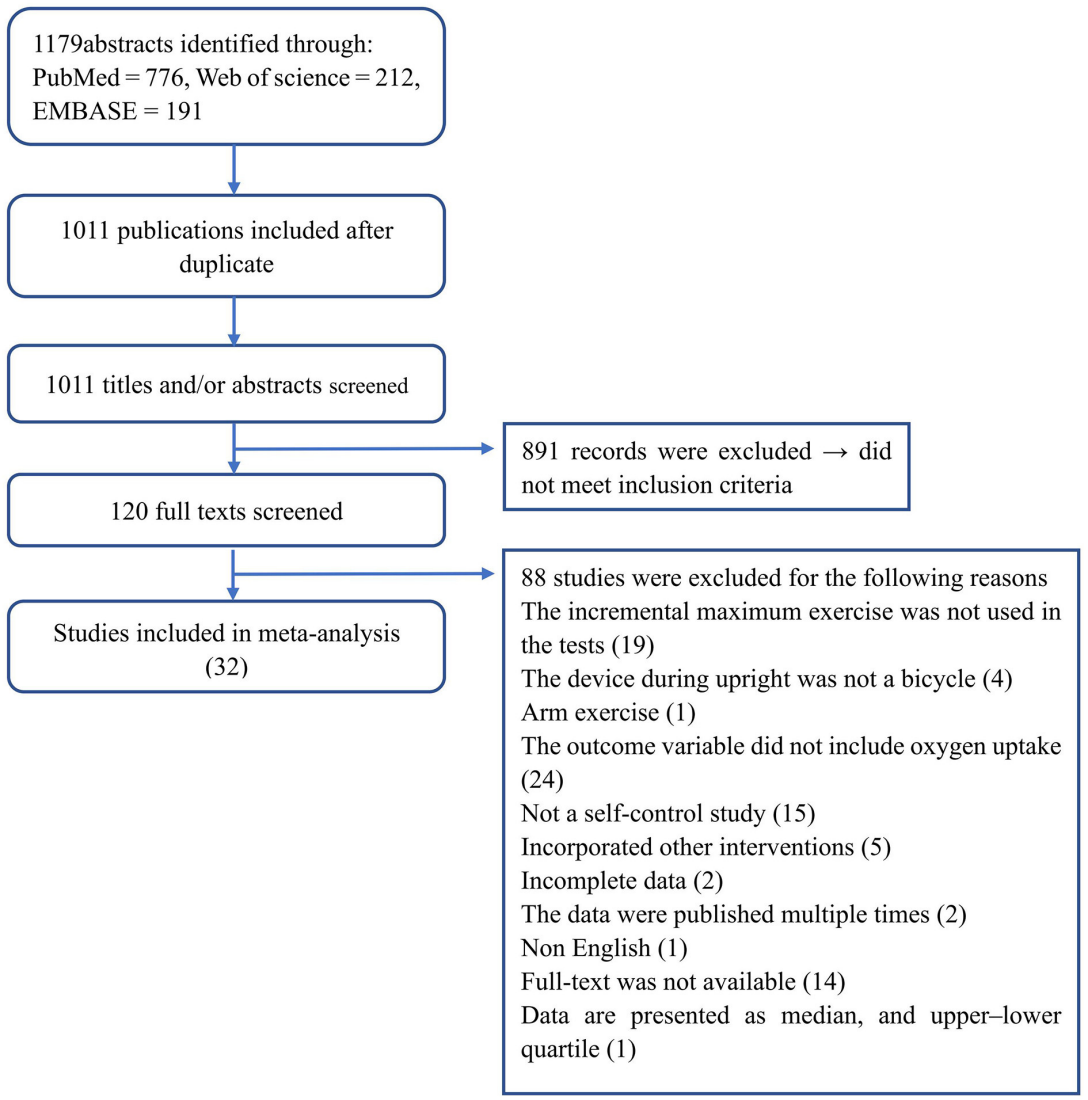
Detailed literature search and study selection process.

The detailed study search and selection process is outlined in Figure 1. In total, we retrieved 1,179 records from the database searches. After excluding duplicates, we screened 1,011 potentially relevant abstracts, and excluded 891 for failing to meet the inclusion criteria. We read the remaining 120 full texts, and deemed 32 self-control trials eligible for systematic review (9, 10, 12–41).

### Methodological Quality of the Included Studies

The quality of the included studies is shown in Table 1. Only nine studies (28%) clarified the participants' inclusion and exclusion criteria. Most of the studies (*n* = 21, 66%) clarified the exercise protocol, but only about half (*n* = 14, 44%) clarified the body position during exercise. Twenty studies (62.5%) mentioned the use of randomization methods to determine the sequence of positions used in the CPET. In addition, the interval between the two tests was similar in 18 (56%) of the studies. Although the VO_2peak_ was clarified in 14(44%) studies, VO_2peak_ was judged to be valid in 26 (81%) studies where the criteria for verification of maximal effort were stated explicitly.

**Table 1:** Methodological quality evaluation for included studies (*n* = 32).

**References**	1. Participant's characteristics clarified?^*^	2. Test protocol clarified?^*^	3. Test positions clarified?^*^	4. Between tests period similar?^*^	5. Test order randomized?^*^	6. VO_**2peak**_ clarified?^*^	7. VO_**2peak**_ test valid?^*^
Ade et al. (15)	NS	Y	Y	N	Y	Y	Y
Armour et al. (16)	NS	NS	N	N	Y	N	Y
Bonzheim et al. (10)	Y	Y	Y	Y	Y	N	Y
Chesler and Stein (17)	Y	Y	Y	Y	Y	N	Y
Cornelis and Buys (18)	Y	Y	N	N	Y	Y	Y
DiMenna et al. (19)	N	Y	NS	N	N	Y	N
Egana et al. (20)	NS	Y	Y	N	Y	N	Y
Egana et al. (21)	N	Y	Y	Y	Y	Y	Y
Faulkner et al. (22)	Y	NS	N	Y	Y	N	Y
Forbregd et al. (23)	NS	Y	Y	Y	Y	Y	Y
Forton et al. (24)	NS	NS	Y	Y	Y	N	Y
Goldstein et al. (25)	NS	Y	N	Y	Y	N	Y
Greenleaf et al. (26)	N	NS	N	Y	N	Y	Y
Hughson et al. (27)	NS	NS	N	N	N	N	N
Hughson et al. (28)	NS	Y	NS	N	N	Y	Y
Jones et al. (29)	N	Y	Y	Y	N	Y	Y
Koga et al. (30)	NS	Y	NS	N	N	N	Y
Kramer et al. (9)	Y	NS	N	Y	N	N	N
Leyk et al. (31)	NS	Y	Y	Y	Y	N	Y
Magder et al. (32)	NS	NS	N	N	N	Y	N
May et al. (13)	Y	Y	N	N	Y	N	Y
Mizumi et al. (14)	Y	Y	Y	N	N	Y	Y
Pedersen et al. (33)	NS	NS	N	Y	Y	N	Y
Quinn et al. (12)	NS	Y	NS	Y	Y	N	Y
Rowland et al. (34)	NS	Y	NS	Y	N	Y	Y
Schulman et al. (35)	Y	NS	N	Y	Y	N	Y
Tempest et al. (36)	NS	NS	Y	N	Y	Y	Y
Terkelsen et al. (37)	NS	Y	N	N	Y	Y	N
Walsh-Riddle et al. (38)	NS	Y	Y	Y	Y	N	Y
Welbergen and Clijsen (39)	NS	Y	Y	Y	N	N	N
Yamada and Sumio (40)	Y	Y	Y	Y	N	Y	Y
Zhao et al. (41)	NS	NS	NS	N	Y	N	Y

*VO_2peak_, peak oxygen uptake; Y, Yes; N, No; NS, Not sure*.

* *Rating criteria details are listed in Supplementary Materials S3*.

### Characteristics of the Included Studies

Details of the participants' characteristics are listed in Table 2. 546 participants were included in the meta-analysis, of which about 62% were male. 21 studies included only healthy people, 9 studies included patients with cardiopulmonary disease, and 2 studies included both the healthy and the patients with cardiopulmonary disease. The ages of the included population ranged from 9 to 72 years old. There were four articles on children (13, 23, 25, 34), one on both adults and children (41), and the remaining 27 articles were on adults.

**Table 2:** Characteristics of participants for the included studies (*n* = 32).

**References**	**Population**	**Participants (*n*)**	**Males (*n*)**	**Age, years**	**BMI (kg/m^**2**^)/Body mass (kg)**
				**Mean ± SD (range)**	**Mean ± SD**
Ade et al. (15)	Healthy	22	22	25 ± 3	23.8 ± 17.6/75.0 ± 17.6
Armour et al. (16)	HF	9	7	61.9 ± 6.1	29.1 ± 2.9/NA
	Healthy	10	6	63.8 ± 4.6	26.5 ± 3.1/NA
Bonzheim et al. (10)	CAD	14	14	60 ± 6	NA/85 ± 11
Chesler and Stein (17)	Healthy	21	0	39 ± 6 (30–50)	23.4 ± 0.43/66.2 ± 1.7
Cornelis and Buys (18)	Healthy	12	8	21.6 ± NA (21–24)	22.0 ± 1.2/NA
DiMenna et al. (19)	Healthy	8	8	35 ± 13	NA/80.3 ± 6.7
Egana et al. (20)	Healthy	22	11	25.1 ± 4.72	NA/67.9 ± 14.07
Egana et al. (21)	Healthy	10	10	24 ± 4	NA/74.4 ± 6.9
Faulkner et al. (22)	Healthy	17	17	24.6 ± 4.3	NA/76.5 ± 8.7
Forbregd et al. (23)	Healthy	31	NA	(9–15)	18.28 ± 2.4/NA
Forton et al. (24)	Healthy	26	13	23 ± 2	NA/67 ± 11
Goldstein et al. (25)	Fontan	29	18	13.4 ± 2.6	19.2 ± 3/NA
	Healthy	16	9	12.7 ± 4.9	19.6 ± 5.1/NA
Greenleaf et al. (26)	Healthy	4	4	38 ± 8 (26–45)	73.7 ± 7.8/NA
Hughson et al. (27)	Healthy	8	7	22.6 ± 0.9	NA/73.3 ± 2.8
Hughson et al. (28)	Healthy	12	12	22 ± 3	NA/74.6 ± 3.4
Jones et al. (29)	Healthy	8	8	24 ± 7	NA/75.0 ± 5.8
Koga et al. (30)	Healthy	9	8	23.8 ± 9.2	NA/65.8 ± 10.6
Kramer et al. (9)	HF	14	14	60 ± NA (48–72)	NA/NA
Leyk et al. (31)	Healthy	9	7	26 ± 6	NA/71 ± 8
Magder et al. (32)	CAD	8	8	59.1 ± 5.6 (49–66)	25.5 ± 1.8/76.75 ± 2.76
May et al. (13)	Healthy	80	40	13.1 ± 2.3 (8.4-17.8)	NA/49.3 ± 14.2
Mizumi et al. (14)	CTEPH	17	5	58 ± 14	NA/NA
Pedersen et al. (33)	Healthy	8	8	22 ± NA (19–27)	NA/73 ± NA
Quinn et al. (12)	Cardiac disease	9	NA	61.5 ± 8.8 (46–73)	NA/72.7 ± 14.4
Rowland et al. (34)	Healthy	13	13	12.5 ± 1.4 (10.3-14.8)	NA/45.5 ± 10.5
Schulman et al. (35)	Hypertension	20	10	55 ± 5	NA/NA
Tempest et al. (36)	Healthy	12	6	26.2 ± 3.0	NA/72.7 ± 9.1
Terkelsen et al. (37)	Healthy	10	5	22 ± NA (19–25)	NA/68.0 ± 3.3
Walsh-Riddle et al. (38)	Hypertension	20	10	47.9 ± NA (34–62)	NA/NA
Welbergen and Clijsen (39)	Healthy	6	6	28 ± 5 (23–34)	NA/83 ± 9
Yamada and Sumio1999 (40)	AMI	19	19	55.3 ± 7.8	NA/64.8 ± 6.8
Zhao et al. (41)	PE	13	11	19 ± 6 (10–31)	NA/56 ± 10

*SD, standard deviation; BMI, body mass index; HF, heart failure; CAD, coronary artery disease; Fontan, Single Ventricle Receiving Fontan Palliation; CTEPH, chronic thromboembolic pulmonary hypertension; AMI, Acute Myocardial Infarction; PE, pectus excavatum. NA, not available*.

Details on the exercise protocols used in the included studies are summarized in Table 3. All of the included studies used the continuous incremental exercise program, except for Quinn's study (12). The angle between the upper body and the horizontal in the supine position ranged from −6 to 65°. In some studies, there was only one position and one test for supine exercise, while in other studies, supine exercise contained multiple positions and multiple tests. For example, Egaña's study showed that the angles between the upper body and the horizontal plane in the supine position included zero, 15 and 30° (21). The interval between the two tests was within 1 month in all studies, except for May's and Mizumi's (13, 14).

**Table 3:** Exercise protocol for the included studies (*n* = 32).

**References**	**Angle^a^, degrees**	**Protocol**	**Starting load**	**Cadence**	**Increments**
Ade et al. (15)	−6	Continuous	20 W	60 rpm	25 W/min
Armour et al. (16)	NA	Continuous	0 W	NA	25 W/3 min
Bonzheim et al. (10)	65	Continuous	25 W	50 rpm	25 W/2 min
Chesler and Stein (17)	0	Continuous	0 W	50 rpm	25 W/2 min
Cornelis and Buys (18)	NA	Continuous	40-75 W	60-70 rpm	25 W or 30 W/min
DiMenna et al. (19)	0	Continuous	0 W	80 rpm	30 W/min
Egana et al. (20)	0	Continuous	M/F:60/30 W	60 rpm	M/F: 30 W/3 min
Egana et al. (21)	0 and 15 and 30	Continuous	60 W	60 rpm	30 W/3 min until 180 W, then 15 W/min
Faulkner et al. (22)	NA	Continuous	U/Rec:60/30 W	NA	U/REC: 1 W/5 s
Forbregd et al. (23)	0 and 45	Continuous	20 W	60 rpm	2 W/5 s
Forton et al. (24)	0 and 35	Continuous	M/F:60/30 W	NA	M/F: 30/20 W/min
Goldstein et al. (25)	NA	Continuous	200 kg·m/min	60-70 rpm	See the original for details
Greenleaf et al. (26)	NA	Continuous	0 W	NA	See the original for details
Hughson et al. (27)	0	Continuous	NA	NA	15 W/min
Hughson et al. (28)	NA	Continuous	25 W	60 rpm	20 W/min
Jones et al. (29)	0	Continuous	0 W	80-85 rpm	30 W/min
Koga et al. (30)	0	Continuous	0 W	60 rpm	25 W/min
Kramer et al. (9)	NA	Continuous	200 kpm/min	NA	100 kpm/min/3 min
Leyk et al. (31)	0	Continuous	20 W	1 Hz	20 W*5 min and 80 W*5 min, then 10 W/30 s until exhaustion
Magder et al. (32)	NA	Continuous	20 W	NA	10 W/min
May et al. (13)	NA	Continuous	0.25*body weight (kg)	50-60 rpm	0.25*body weight(kg)/min
Mizumi et al. (14)	0	Continuous	10 W	60 rpm	10 W/min
Pedersen et al. (33)	NA	Continuous	160 W	80 rpm	See the original for details
Quinn et al. (12)	0 and 35	Discontinuous	150 kg·m/min	50 rpm	150 kg·m/min
Rowland et al. (34)	0	Continuous	25 W	50 rpm	25 W/3min
Schulman et al. (35)	NA	Continuous	25 W	NA	25 W/3min
Tempest et al. (36)	45 and 65	Continuous	NA	70 rpm	20 W/min
Terkelsen et al. (37)	NA	Continuous	50 W	60 rpm	50 W/3min
Walsh-Riddle et al. (38)	45	Continuous	40 W	50 rpm	40 W*3min and 80 W*3min and 120 W*3min, then 20 W/min
Welbergen and Clijsen (39)	45	Continuous	100 W	90 rpm	100 W*3min and 200 W*3min, then give maximal effort
Yamada and Sumio (40)	0	Continuous	10 W	50 rpm	10 W/min
Zhao et al. (41)	0	Continuous	0 W	NA	15-30 W/min

a *Angle between upper body and horizontal plane in supine position*.

*M, male; F, female; U, upright; REC, recumbent; W, watt; Kpm, Kilopounds; min, minute; s, second; rpm, revolutions per minute; NA, not available*.

### Comparison of VO_2peak_ Between Positions

**Figure 2:**
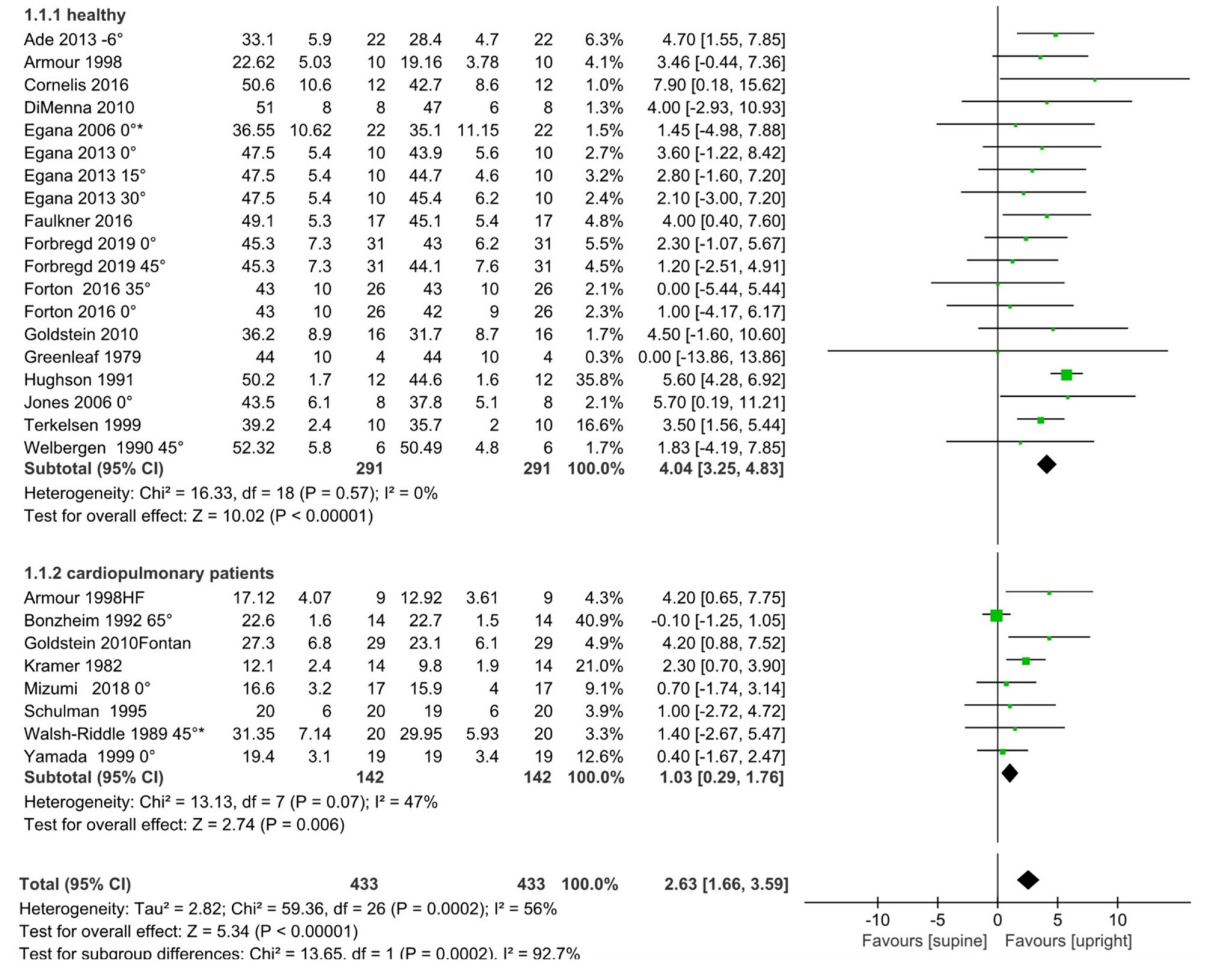
Meta-analysis results for relative VO_2peak_ (ml/kg/min), including overall pooled effects and subgroup effects of the healthy and the cardiopulmonary disease patients. HF, heart failure; Fontan, Single Ventricle Receiving Fontan Palliation. A degree (°) symbol after the reference refers to the angle between upper body and horizontal plane in the supine position. A reference without a degree indicates that the details were unavailable in the original text. An asterisk means that the original text's grouping data are combined according to the formula in Cochrane 7.7, because the angle between the upper body and the horizontal plane is the same as in the supine position.

In terms of the relative VO_2peak_, our pooled results showed that the upright VO_2peak_ was higher than the supine VO_2peak_ (relative VO_2peak_: MD = 2.63 ml/kg/min, 95% CI: 1.66, 3.59, *I*^2^ = 56%, *p* < 0.05; Figure 2). In the healthy subgroup, the upright VO_2peak_ remained higher than supine and the effect size was larger without heterogeneity (relative VO_2peak_: MD = 4.04 ml/kg/min, 95% CI: 3.25, 4.83, *I*^2^ = 0%; Figure 2). Similarly, in patients with cardiopulmonary disease, upright VO_2peak_ was also higher than supine, however, the effect size was lower compared to the healthy subgroup (relative VO_2peak_: MD = 1.03 ml/kg/min, 95% CI: 0.29, 1.76, *I*^2^ = 47%; Figure 2).

**Figure 3:**
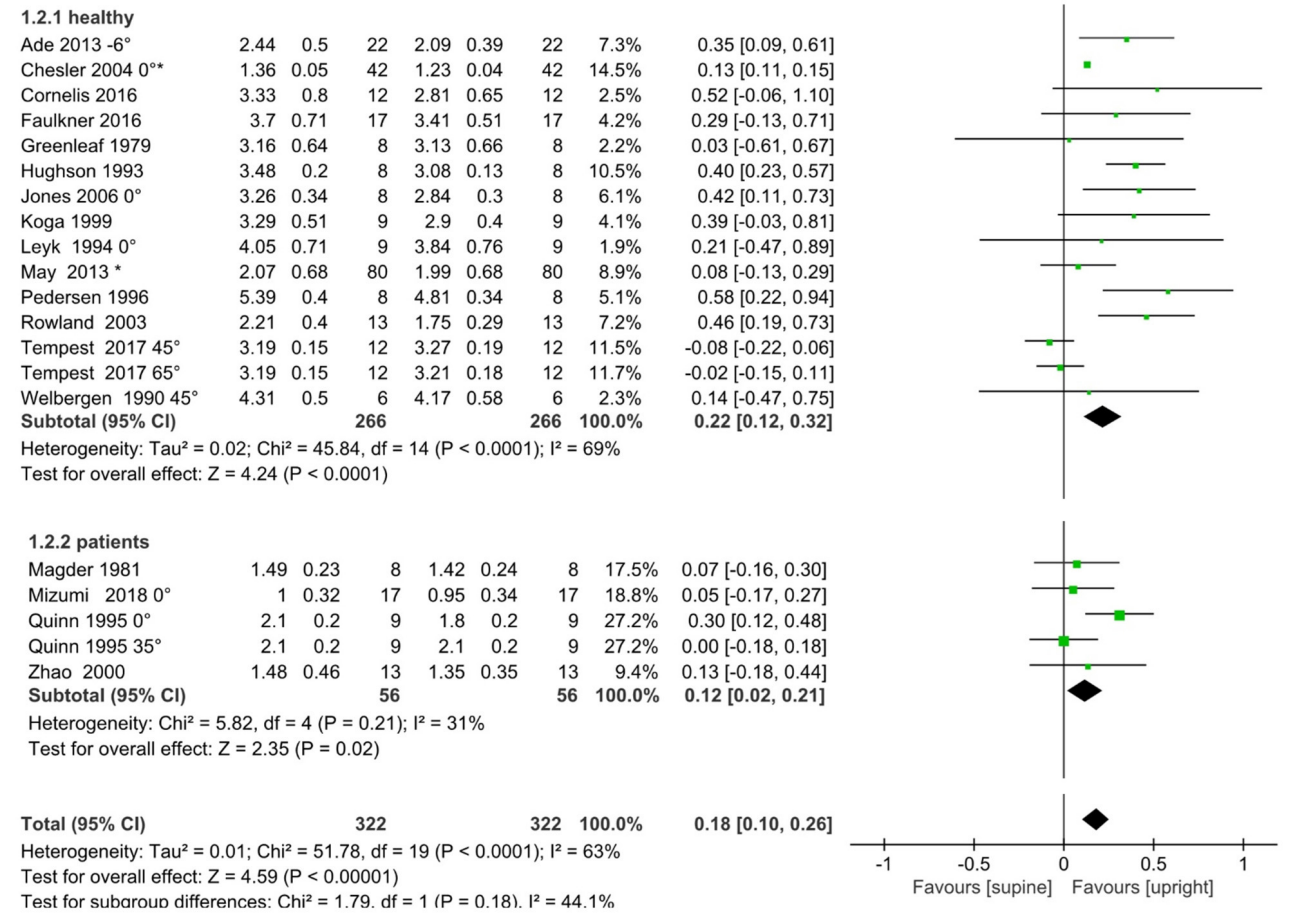
Meta-analysis results for absolute VO_2peak_ (L/min), including overall pooled effects and subgroup effects of the healthy and the cardiopulmonary disease patients. A degree (°) marker after the reference refers to the angle between upper body and horizontal plane in the supine position. References without a degree symbol indicates that the details were unavailable in the original text. An asterisk means that the original text's grouping data are combined according to the formula in Cochrane 7.7, because the angle between upper body and horizontal plane is the same in the supine position.

In terms of the absolute VO_2peak_, our pooled results also showed that the upright VO_2peak_ was higher than the supine VO_2peak_ (absolute VO_2peak_: MD = 0.18 L/min, 95% CI: 0.10, 0.26, *I*^2^ = 63%, *p* < 0.05; Figure 3). Again, the effect size was higher in the healthy subgroup (absolute VO_2peak_: MD = 0.22 L/min, 95% CI: 0.12, 0.32, *I*^2^ = 69%; Figure 3), but lower in the cardiopulmonary disease subgroup (absolute VO_2peak_: MD = 0.12 ml/kg/min, 95% CI: 0.02, 0.21 *I*^2^ = 31%; Figure 3).

### Discussion

The purpose of this paper is to evaluate the effect of body position on VO_2peak_ in the CPET *via* meta-analysis. The results indicate that the VO_2peak_ measured by CPET in the upright position was higher than that of the supine position during an incremental cycling exercise test. Moreover, this difference exists in both healthy people and patients with cardiopulmonary disease.

Physiologic shifts in hemodynamic distribution occur with along with changes from supine to upright postures. This hemodynamic shift alters the preload to the right ventricle which subsequently affects preload to the left ventricle and the Frank-Starling mechanism associated with ventricular contraction and stroke volume. Thadani and Parker. have reported hemodynamic differences between the supine and upright positions in normal subjects. They found higher heart rates with lower left ventricular filling pressures and stroke volume index in the upright position, both at rest and during exercise. However, they noted no differences in cardiac index or peak work load between the two modes of exercise (42). Kramer's study of heart failure patients also showed significantly higher right atrial pressure in the supine position, but no differences in cardiac index or stroke index between the two positions. Further, these authors found significantly lower VO_2peak_ in the supine position (9). Moreover, in chronic thromboembolic pulmonary hypertension patients, Mizumi's study showed that CO at rest in the supine position was significantly higher than that in the upright. However, CO at peak exercise was comparable between the two positions, even though VO_2peak_ in the supine position tends to be lower than that of the upright (14). These studies suggest that increased ventricular preload caused by postural changes does not necessarily lead to an increase in stroke volume or CO as a key mechanism to facilitate increased oxygen uptake in the upright posture. In contrast, these data suggest that VO_2peak_ in the supine position may be limited by factors other than the effect of central hemodynamic shifts on CO.

According to the Fick principle, VO_2_ = CO ^*^ C(a-v) O_2_ (arteriovenous oxygen difference) (43). Therefore, the VO_2_ is influenced by both central and peripheral components, including CO and C(a-v) O_2_, during exercise. Studies have shown that VO_2peak_ in the upright position is higher than that in the supine position, and accompanied by higher C(a-v) O_2_, while there is no difference in cardiac index between the two positions (9, 44). The change in C(a-v) O_2_ is influenced by both muscle's ability to consume oxygen and the amount of muscle mass involved in the activity. The oxygen uptake in the muscles is determined by factors such as the amount of muscle work, perfusion pressure, blood flow and mitochondrial density and activity. With regards to muscle mass, the upright position requires greater muscle mass involvement, and thus more physiologic work. Research by Bouillon et al. has demonstrated that during upright cycling, the maximum voluntary isometric contraction of the gluteus maximus, gluteus medius, biceps femoris, lateral head of gastrocnemius, anterior tibialis, rectus femoris, lumbar erector spinalis and rectus abdominis exceed those measured in the semi-reclining position (45). Furthermore, when standing upright, the distribution of blood in the lower extremity vein increases due to increased orthostatic tension. Additionally, the arteries' diameter is wider than in the supine position, and the blood flow velocity is slower. This is conducive to the diffusion of more oxygen from hemoglobin to myoglobin (46). Furthermore, Eiken found that exposing working legs to sub-atmospheric pressure can increase perfusion pressure. This can improve motor ability in the supine position, and simulate upright exercise in normal gravity (47, 48). Further, several studies have used near-infrared spectroscopy illuminate the oxyhemoglobin relationship in the periphery during exercise. These studies suggest that muscle oxygen absorption capacity is higher in the upright position (49–51). Therefore, the C(a-v) O_2_ during peak exercise is higher in the upright position, and this may contribute to the increased VO_2peak_ noted in the upright position.

In addition, studies have shown that the increased venous return in the supine position is conducive to increasing the fluid shift to the pulmonary circulation and interstitium. This may compress small airways and/or blood vessels, unbalancing the overall pulmonary V/Q ratio and reducing ventilatory efficiency (52). Bryan et al. (5) demonstrated that the change from an upright position to the supine position in healthy individuals results in a decrease in the V/Q of the entire lung from 0.83 to 0.76. Further, Sandoval et al. have shown that even in the resting state, the change in body position causes significant changes in alveolar-arterial oxygen partial pressure difference in patients with Eisenmenger syndrome. This decreases oxygen saturation in the supine position (53). In contrast, other studies have shown that the change from an upright position to a supine does not cause significant reduction in ventilatory efficiency in healthy people, or even in patients with stable heart failure (16, 18, 24, 37). As such, the extent to which these components affect peak oxygen uptake in the supine position, and contribute to the lower values when compared to those measured in the upright position, remains unclear.

### Limitations

It is important to acknowledge limitations associated with this study. First, the sample sizes of most studies in this meta-analysis were very small. Second, since this research is a self-control study, it is difficult to blind participants to the exercise condition, and therefore difficult to prevent unintentional bias. Third, the difference in VO_2peak_ between the upright position and the supine position may vary among patient groups, and the data for patients with cardiopulmonary disease was insufficient. Therefore, we could not conduct subgroup analysis according to specific disease types. In the future, with the increase in research data from patients with cardiopulmonary disease, subgroup analysis could be conducted according to specific disease types. Lastly, this analysis was unable to account for different supine position body angles. The body position of research participants should be clarified in future clinical studies to facilitate subgroup analysis.

## Conclusion

VO_2peak_ in the upright position is higher than that measured in the supine position, in both healthy subjects and patients with cardiopulmonary disease. However, additional verification is needed to determine whether this difference has clinical significance. Like bicycle and treadmill exercise modalities, both supine and upright exercise modalities can be applied for differential diagnosis, surgical risk assessment, prognosis, and to evaluate overall exercise capacity and therapeutic effects of a variety of clinical interventions. Researchers should consider these differences across exercise modalities, and choose the form of exercise which best suits their clinical requirements.

## Data Availability Statement

The raw data supporting the conclusions of this article will be made available by the authors, without undue reservation.

## Author Contributions

XW and WJ contributed to the conception and design of the study. XW and CL contributed to the data acquisition, analysis, and interpretation. XW drafted the first version of the manuscript and revised it based on other authors' contributions. All authors contributed important intellectual content to the manuscript's critical revision, read, and approved the final manuscript.

## Funding

This work was supported by the Guangdong Provincial Key Laboratory of Chinese Medicine for Prevention and Treatment of Refractory Chronic Diseases (grant number: 2018B030322012); the General Research Fund of Traditional Chinese Medicine Science and Technology from Guangdong Provincial Hospital of Chinese Medicine (YN2018ML02); and the Clinical Research Funding of Traditional Chinese Medicine Science and Technology (Project 1010) from Guangdong Provincial Hospital of Chinese Medicine (YN10101910).

## Conflict of Interest

The authors declare that the research was conducted in the absence of any commercial or financial relationships that could be construed as a potential conflict of interest.

## Publisher's Note

All claims expressed in this article are solely those of the authors and do not necessarily represent those of their affiliated organizations, or those of the publisher, the editors and the reviewers. Any product that may be evaluated in this article, or claim that may be made by its manufacturer, is not guaranteed or endorsed by the publisher.

## Abbreviations

C(a-v) O_2_: arteriovenous oxygen difference
CO: cardiac output
CPET: cardiopulmonary exercise test
SD: standard deviation
MD: mean difference
VO_2peak_: peak oxygen uptake
V/Q: Ventilation/perfusion ratio.
