# Supplementary material for: Differences in Peak Oxygen Uptake in Bicycle Exercise Test Caused by Body Positions: A Meta-Analysis
Source: Front Cardiovasc Med. 2021 Oct 11;8:734687. doi: 10.3389/fcvm.2021.734687 (PMC8542763; doi:10.3389/fcvm.2021.734687)
Supplement: Supplementary file 1 [file Data_Sheet_1.docx]

**Supplementary Materials**

**S1.** **PRISMA 2020 Checklist**

| **Section and Topic** | **Item #** | **Checklist item** | **Location where item is reported** |
| --- | --- | --- | --- |
| **TITLE** | | |  |
| Title | 1 | Identify the report as a systematic review. | #1 |
| **ABSTRACT** | | |  |
| Abstract | 2 | See the PRISMA 2020 for Abstracts checklist. | #2 |
| **INTRODUCTION** | | |  |
| Rationale | 3 | Describe the rationale for the review in the context of existing knowledge. | #3 |
| Objectives | 4 | Provide an explicit statement of the objective(s) or question(s) the review addresses. | #3 |
| **METHODS** | | |  |
| Eligibility criteria | 5 | Specify the inclusion and exclusion criteria for the review and how studies were grouped for the syntheses. | #4 |
| Information sources | 6 | Specify all databases, registers, websites, organisations, reference lists and other sources searched or consulted to identify studies. Specify the date when each source was last searched or consulted. | #3 |
| Search strategy | 7 | Present the full search strategies for all databases, registers and websites, including any filters and limits used. | S2 |
| Selection process | 8 | Specify the methods used to decide whether a study met the inclusion criteria of the review, including how many reviewers screened each record and each report retrieved, whether they worked independently, and if applicable, details of automation tools used in the process. | #4 |
| Data collection process | 9 | Specify the methods used to collect data from reports, including how many reviewers collected data from each report, whether they worked independently, any processes for obtaining or confirming data from study investigators, and if applicable, details of automation tools used in the process. | #4 |
| Data items | 10a | List and define all outcomes for which data were sought. Specify whether all results that were compatible with each outcome domain in each study were sought (e.g. for all measures, time points, analyses), and if not, the methods used to decide which results to collect. | #4 |
|  | 10b | List and define all other variables for which data were sought (e.g. participant and intervention characteristics, funding sources). Describe any assumptions made about any missing or unclear information. | #4 |
| Study risk of bias assessment | 11 | Specify the methods used to assess risk of bias in the included studies, including details of the tool(s) used, how many reviewers assessed each study and whether they worked independently, and if applicable, details of automation tools used in the process. | - |
| Effect measures | 12 | Specify for each outcome the effect measure(s) (e.g. risk ratio, mean difference) used in the synthesis or presentation of results. | #4-5 |
| Synthesis methods | 13a | Describe the processes used to decide which studies were eligible for each synthesis (e.g. tabulating the study intervention characteristics and comparing against the planned groups for each synthesis (item #5)). | #4-5 |
|  | 13b | Describe any methods required to prepare the data for presentation or synthesis, such as handling of missing summary statistics, or data conversions. | #4-5 |
|  | 13c | Describe any methods used to tabulate or visually display results of individual studies and syntheses. | #4-5 |
|  | 13d | Describe any methods used to synthesize results and provide a rationale for the choice(s). If meta-analysis was performed, describe the model(s), method(s) to identify the presence and extent of statistical heterogeneity, and software package(s) used. | #4-5 |
|  | 13e | Describe any methods used to explore possible causes of heterogeneity among study results (e.g. subgroup analysis, meta-regression). | #5 |
|  | 13f | Describe any sensitivity analyses conducted to assess robustness of the synthesized results. | #5 |
| Reporting bias assessment | 14 | Describe any methods used to assess risk of bias due to missing results in a synthesis (arising from reporting biases). | - |
| Certainty assessment | 15 | Describe any methods used to assess certainty (or confidence) in the body of evidence for an outcome. | #4 |
| **RESULTS** | | |  |
| Study selection | 16a | Describe the results of the search and selection process, from the number of records identified in the search to the number of studies included in the review, ideally using a flow diagram. | Fig. 1 |
|  | 16b | Cite studies that might appear to meet the inclusion criteria, but which were excluded, and explain why they were excluded. | Fig. 1 |
| Study characteristics | 17 | Cite each included study and present its characteristics. | Table 1-3 |
| Risk of bias in studies | 18 | Present assessments of risk of bias for each included study. | - |
| Results of individual studies | 19 | For all outcomes, present, for each study: (a) summary statistics for each group (where appropriate) and (b) an effect estimate and its precision (e.g. confidence/credible interval), ideally using structured tables or plots. | Fig. 2-3 |
| Results of syntheses | 20a | For each synthesis, briefly summarise the characteristics and risk of bias among contributing studies. | - |
|  | 20b | Present results of all statistical syntheses conducted. If meta-analysis was done, present for each the summary estimate and its precision (e.g. confidence/credible interval) and measures of statistical heterogeneity. If comparing groups, describe the direction of the effect. | Fig. 2-3 |
|  | 20c | Present results of all investigations of possible causes of heterogeneity among study results. | Fig. 2-3 |
|  | 20d | Present results of all sensitivity analyses conducted to assess the robustness of the synthesized results. | Fig. 2-3 |
| Reporting biases | 21 | Present assessments of risk of bias due to missing results (arising from reporting biases) for each synthesis assessed. | - |
| Certainty of evidence | 22 | Present assessments of certainty (or confidence) in the body of evidence for each outcome assessed. | - |
| **DISCUSSION** | | |  |
| Discussion | 23a | Provide a general interpretation of the results in the context of other evidence. | #6-7 |
|  | 23b | Discuss any limitations of the evidence included in the review. | #7 |
|  | 23c | Discuss any limitations of the review processes used. | #7 |
|  | 23d | Discuss implications of the results for practice, policy, and future research. | #8 |
| **OTHER INFORMATION** | | |  |
| Registration and protocol | 24a | Provide registration information for the review, including register name and registration number, or state that the review was not registered. | #3 & 8 |
|  | 24b | Indicate where the review protocol can be accessed, or state that a protocol was not prepared. | #8 |
|  | 24c | Describe and explain any amendments to information provided at registration or in the protocol. | - |
| Support | 25 | Describe sources of financial or non-financial support for the review, and the role of the funders or sponsors in the review. | #8 |
| Competing interests | 26 | Declare any competing interests of review authors. | #8 |
| Availability of data, code and other materials | 27 | Report which of the following are publicly available and where they can be found: template data collection forms; data extracted from included studies; data used for all analyses; analytic code; any other materials used in the review. | #8 |

**S2. Search strategy**

**PubMed search strategy**

1. “Exercise test” [MeSH Terms])

2. “exercise test*” [All Fields] OR “cardiopulmonary exercise test*” [All Fields] OR “cycle exercise*” [All Fields] OR “ergometer*” [All Fields] OR “CPET” [All Fields] OR “CPX” [All Fields]

3. or 1/2

4. “position*” [All Fields] OR “posture*” [All Fields] OR “supine” [All Fields] OR “recumbent” [All Fields] OR “recline” [All Fields] OR “lean” [All Fields] OR “tilt” [All Fields] OR “clinostatism” [All Fields] OR “decubitus” [All Fields] OR “lie” [All Fields]

5. “erect” [All Fields] OR “upright” [All Fields] OR “orthostatic” [All Fields] OR “sit*" [All Fields])

6. 4 AND 5

7. 3 AND 6

**Web Of Science search strategy**

1. “Exercise test" OR "exercise test*" OR "cardiopulmonary exercise test*" OR "cycle exercise*" OR "ergometer*" OR "CPET" OR "CPX" (Topic)

2. "position*" OR "posture*" OR "supine" OR "recumbent" OR "recline" OR "lean" OR "tilt" OR “clinostating” OR "decubitus" OR "lie" (Topic)

3. "erect" OR "upright" OR "orthostatic" OR "sit*" (Topic)

4. “VO2” OR “oxygen uptake”

5. 1 AND 2 AND 3 AND 4

**Embase search strategy**

1. “exercise test*” OR “cycle exercise*” OR “ergometer*” OR “cpet” OR “cpx”

2. “position*” OR “posture*” OR “supine” OR “recumbent” OR “recline” OR “lean” OR “tilt” OR “clinostating*” OR “decubitus*” OR “lie*”

3. “erect” OR “upright” OR “orthostatic*” OR “sit*”

4. “vo2” OR “oxygen uptake”

5. 1 AND 2 AND 3 AND 4

**S3. Modified Downs and Black checklist. (Down & Black, 1998)**

| **Domain** | **Items** | **Criteria** |
| --- | --- | --- |
| **Population** | 1. **Characteristics of the participants** | **Are the characteristics of the participants included in the study clearly described?**  Inclusion and/or exclusion criteria should be given. For unhealthy participants, the type of disease should be clearly described.  **YES:** both are given.  **No:** none is given.  **Not sure:** only one is given. |
| **Exercise tests** | 1. **Test protocol** | **Are the interventions of interest clearly described?**  The ≥2 test modes that are compared should be clearly described. This entails that both the starting load (W/speed/rpm, ect.) and the increment load and increment duration should be reported.  **Yes:** The test can be repeated by reading the descriptions.  **No:** No information is given.  **Not sure:** Descriptions were given partially; it is hard to repeat the tests based on the information in the paper. |
|  | 1. **Test positions** | **Are the test positions clearly described?**  The test positions that are compared should be clearly described.  **Yes:** The angle between the body and the floor is directly reported; or pictures of the positions were given.  **No:** Only mentioned the word “spine” etc, no detailed descriptions.  **Not sure:** Positions were partially described the, and the angles can be known based on the descriptions. |
|  | 1. **Test time period** | **Is the time period the participants have between tests roughly the same?**  **Yes:** The time between repeated tests is within 2 weeks.  **No:** The time between repeated tests is not mentioned. |
|  | 1. **Test order randomized?** | **Was the test order randomized?**  Studies, which state that the test order was randomized or counter-balanced, should be answered **Yes**. |
| **VO2peak** | 1. **Definition** | **How VO2peak was defined?**  How VO2peak was defined should be described for this item to be rated as a **Yes.**  If it is only mentioned that VO2peak was measure but no definition is added, this item should be rated as a **No**. |
|  | 1. **Validation** | **Was the VO2peak test valid?**  This item should be rated as a **Yes**, if the criteria for verification of maximal effort are explicitly stated. Verification of maximal effort should at least contain one of the following four minimum criteria: 1) respiratory exchange ratio (RER) ≥ 1.05, 2) a subjective rating of perceived exertion (RPE) with a BORG scale score ≥ 15, 3) no increase in VO2 despite further increases in intensity, 4) reaching a maximal heart rate within 10 beats/min of an individual’s age-predicted maximum (calculated as 220 – age-10 for upper-body exercise).  Any studies which did not report on the verification of maximal effort or which include criteria below the above described, should be rated with a **No**. |
